# The impact of anthropogenic activities on antimicrobial and heavy metal resistance in aquatic environments

**DOI:** 10.1128/aem.02317-24

**Published:** 2025-03-12

**Authors:** Md Javed Foysal, Brett A. Neilan, Verlaine Timms

**Affiliations:** 1 School of Environmental and Life Sciences, The University of Newcastle 98494 , Callaghan, New South Wales, Australia; 2 Australian Research Council Centre of Excellence in Synthetic Biology, Macquarie University, Sydney, New South Wales, Australia; Colorado School of Mines, Golden, Colorado, USA

**Keywords:** aquatic environments, bacterial communities, drug resistance, resistance frequency, genomics

## Abstract

This study investigated the prevalence and co-occurrence of antimicrobial (AMR) and metal resistance (MR) in aquatic environments with different human impacts. Metagenomes from pristine, rural and urban sites in Australia were analyzed with AMR ++ and customized binning pipelines. AMR was present in all environments, while MR was mainly in rural and urban samples. AMR gene diversity was higher in rural and urban sites, exhibiting resistance to more antibiotic classes (*n* = 10) than the pristine site (*n* = 4). Metal and multicompound resistance was more frequent in rural (14%) compared to urban samples (5%). Pristine samples lacked multidrug and multicompound resistance and had lower resistance to aminoglycosides and the MLS group. Multiresistance was evidenced by copper and aminocoumarin resistance in rural samples and aminoglycoside and mercury resistance in *Pseudomonas* in all environments. These findings highlight the impact of human activities on AMR and MR spread, emphasizing the need for environmental monitoring and management.

**IMPORTANCE:**

Antimicrobial resistance (AMR) and metal resistance (MR) are critical global health concerns exacerbated by anthropogenic activities. The intricate mechanism underlying the interaction among anthropogenic activities, microbial communities, and resistance remains enigmatic. We developed novel bioinformatic pipelines to unveil this interaction in three aquatic environments. Our findings demonstrate the presence of specific bacterial communities that drive AMR and MR in rural and urban environments. This study underscores the significance of proper agricultural practices, comprehensive monitoring, and management strategies to reduce anthropogenic impacts on environmental resistance.

## INTRODUCTION

Bacterial antimicrobial resistance (AMR) and metal resistance (MR) have prehistoric origins but are now emerging as a major global health concern. Anthropogenic activities accelerate the spread, evolution, and transmission of bacterial AMR from wastewater treatment plants, agricultural lands, and industrial effluents (1). The overuse and unplanned discharge of antibiotics into the environment result in sustained environmental antibiotic pressure. This has led to the widespread distribution of antibiotic resistance genes (ARG) and metal resistance genes (MRG) from bacteria that are continually subjected to this selective pressure (2). Furthermore, the widespread use of antibiotics in human and veterinary healthcare, as well as in fish farming, has contributed to the propagation of bacterial AMR (3). This has led to its presence in agricultural soils and aquatic environments, including ponds, lakes, and rivers (4, 5).

AMR and MR are widely distributed primarily through horizontal gene transfer of mobile genetic elements or vertical gene transfer by the proliferation of resistant species (6). Heavy metal pollution plays a unique role in this problem. It not only allows resistant bacteria to survive in ecosystems and aid in disseminating AMR but is also linked to a higher relative abundance of ARGs, even without antibiotic pollution (7). Therefore, in environments positive for MRGs, the potential for ARG co-occurrence is likely (8, 9). In such environments, the frequency of AMR is significantly higher in MR-containing opportunistic pathogens, such as *Escherichia coli* and *Shigella* (8). Previous studies showed a strong co-occurrence of antibiotic and metal resistance genes in various environmental locations, including agricultural lands (10) and aquatic bodies (11). For example, aquaculture tanks and pond sediments contaminated with metals (Cu–Zn) led to resistance to tetracycline, sulfanilamide, and cefotaxime in *E. coli*, along with copper resistance (11, 12). Furthermore, sulfonamide (*sul*1) resistance was found to be associated with the detection of resistance genes to mercury (*mer*A, *mer*B, *mer*D, *mer*T, and *mer*P) and silver (*sil*A, *sil*B, *sil*C, *sil*E, *sil*P, *sil*R, and *sil*S) in *Thiomonas* and *Lactobacillus* from a fully operational biogas reactor (13). Even relatively low concentrations of heavy metals in the environment can significantly influence the selection and proliferation of AMR (14).

The co-occurrence is also linked to the co-transfer of ARG and MRG into other bacteria in various environments such as the human gut (15), soil (16), and sediment (17) via mobile genetic elements (MGE). To date, most studies have focused on the animal gut, soil, and single-source water bodies such as rivers (18–20). This suggests that further investigation is needed into the co-occurrence of ARGs and MRGs in aquatic environments. Therefore, screening both ARGs and MRGs in resistant bacteria is crucial. This will enhance our understanding and ultimately contribute to preserving environmental and animal health.

Aquatic environments are considered ideal habitats for the persistence of AMR–MR due to contamination from various sources such as sewage discharge, agricultural run-off, and metal leaching (21). Research focusing on either AMR or MR has revealed their widespread presence in oceans, rivers, lakes, and ponds (22, 23). Recent studies have indicated that Proteobacteria and Cyanobacteria dominate AMR–MR and toxin-producing communities in both rural and urban aquaculture ponds (24). The study highlighted changes in the microbial communities and the emergence of metal-driven AMR and toxins, primarily due to anthropogenic activities and industrial pollution. Proteobacteria and Actinobacteria are known to be the primary hosts for much of the AMR–MR resistance (25), while Cyanobacteria have been identified as potential sources for the spread and acquisition of AMR in aquatic environments (26, 27). Understanding the relationship between human activities, AMR, and MR in aquatic environments is crucial in predicting the prevalence and anticipating further threats of ARG and MRG to aquatic ecosystems.

With recent advancements in high-throughput sequencing, particularly metagenomic sequencing, it has become possible to conduct in-depth analysis of AMR and host–microbial communities in many environments (28, 29). However, there is a lack of sequencing studies exploring the relationship between anthropogenic activities, aquatic environments, AMR, and MR. In our comparative analysis, we used metagenomic data sets from Centennial Park and Ross Wallbridge reserves in New South Wales, as well as Shark Bay in Western Australia. To understand the anthropogenic impacts on the abundance of AMR and MR in water bodies, our objectives were to (i) profile microbial communities, ARG, and MRG in pristine, rural, and urban water bodies and (ii) identify the co-occurrence of AMR and MR in samples affected by varying levels of human activities. The findings of this study provide valuable insights into the relationship between ARG and MRG and their co-occurrences in pristine, rural, and urban aquatic systems.

## RESULTS

### Sequence statistics and assembly data

After quality trimming, the metagenomic data from 35 samples yielded 2,836.5 million reads, averaging 82.1 million reads per sample (ranging from 72.5 to 98.9 million). Urban samples averaged 82 million reads per sample, rural samples 81 million reads, and pristine samples from Shark Bay also averaged 82 million reads (Table S1). Metagenome assembly produced 58,315,136 contigs, averaging 166,146 per data set (ranging from 84,843 to 695,888). The average contig length was higher for samples collected in December and January (483 ± 73 Kbp) than those collected from February to May (371 ± 37 Kbp).

### Taxonomic diversity in pristine, rural, and urban environments

On average, 36.7% of the reads from the 35 samples were taxonomically assigned using Kraken v2. Among the classified reads, Proteobacteria were the most prevalent, comprising 85.6% of the microbial communities in pristine samples, 67.7% in urban samples, and 46.5% in rural samples. Cyanobacteria were most abundant in rural samples, with a 51.2% abundance among the classified reads, followed by urban samples at 21.1%, while pristine samples showed limited cyanobacterial presence, at just 0.4%.

**Fig 1:**
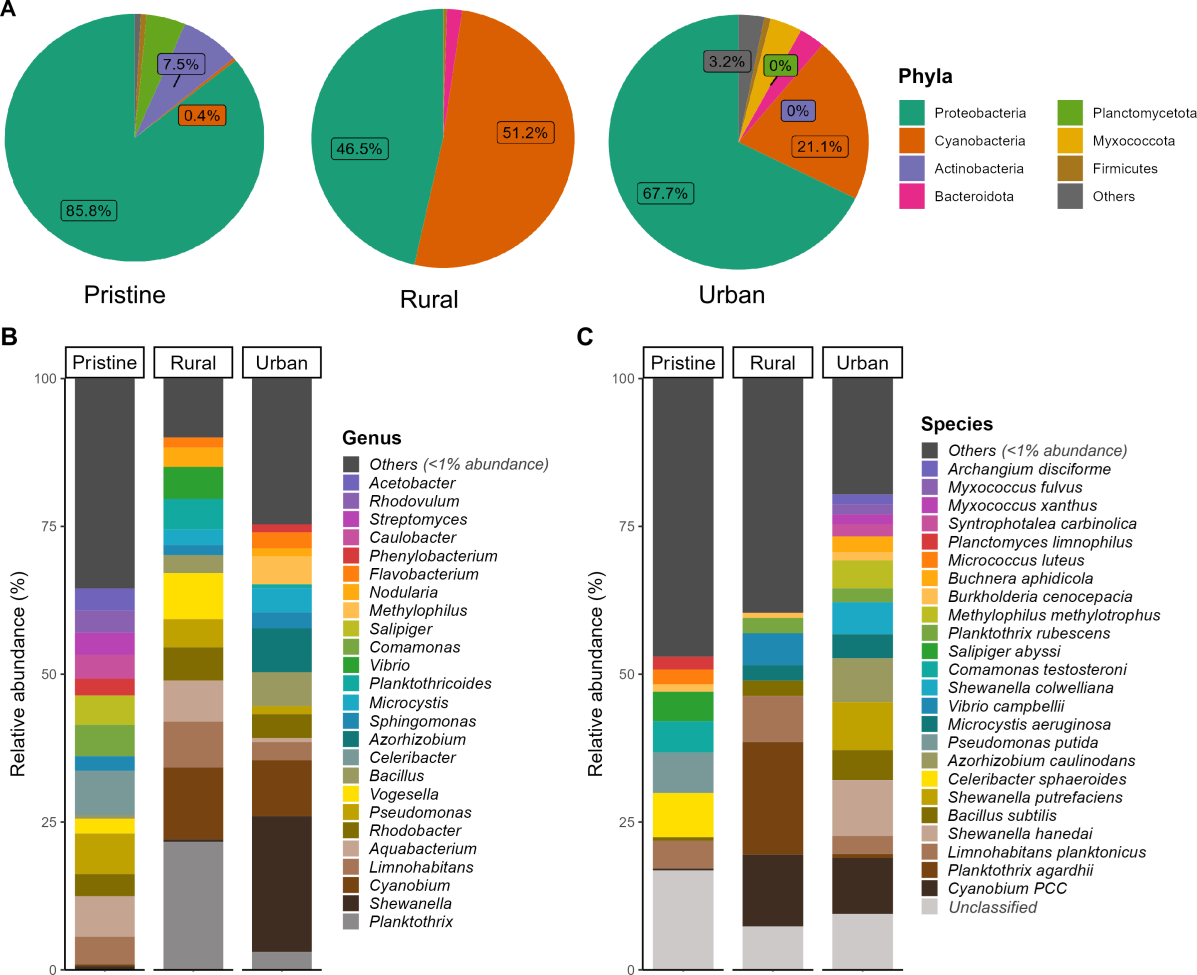
Bacterial composition in pristine, rural, and urban environments. Relative abundance of bacteria at phylum (**A**), genus (**B**), and species (**C**) levels. The pie charts show phyla with more than 1% relative abundance in each environment. Low abundance species (<1%) were grouped as “Others” for genus- and species-level analysis. Unidentified, uncultured, and ambiguous taxa were grouped as “unclassified” bacteria.

In pristine samples, Proteobacteria (85.8%), Actinobacteria (7.5%), and Plancmycetota (5.2%) constituted over 98% of the classified bacterial communities. These samples primarily included *Limnohabitans*, *Aquabacterium*, *Rhodobacter*, *Pseudomonas*, *Vogesella*, *Celeribacter*, *Sphingomonas*, *Comamonas*, *Salipiger*, and *Phenylobacterium* and were largely free from pathogens or toxic bacteria. Conversely, in rural samples, the majority of reads (97%) were classified as Cyanobacteria (51.2%) and Proteobacteria (46.5%). Bacteriodota and Myxococcota were exclusive to urban samples (5%) (Fig. 1A). *Planktothrix* and *Shewanella* dominated rural and urban samples, respectively (Fig. 1B). *Pseudomonas* was distributed uniformly across three different environments (Fig. S1-S4). *Cyanobium* exhibited a uniform relative abundance across both rural and urban samples (Fig. 1B). At the species level, the microbial community was highly diverse across all samples, with only *Rhodobacter* sp., and *Limnohabitans planktonicus* being common to all habitats (Fig. 1C, Fig. S1 to S4).

### Frequency, types, and classes of AMR-MR in pristine, rural, and urban environments

**Fig 2:**
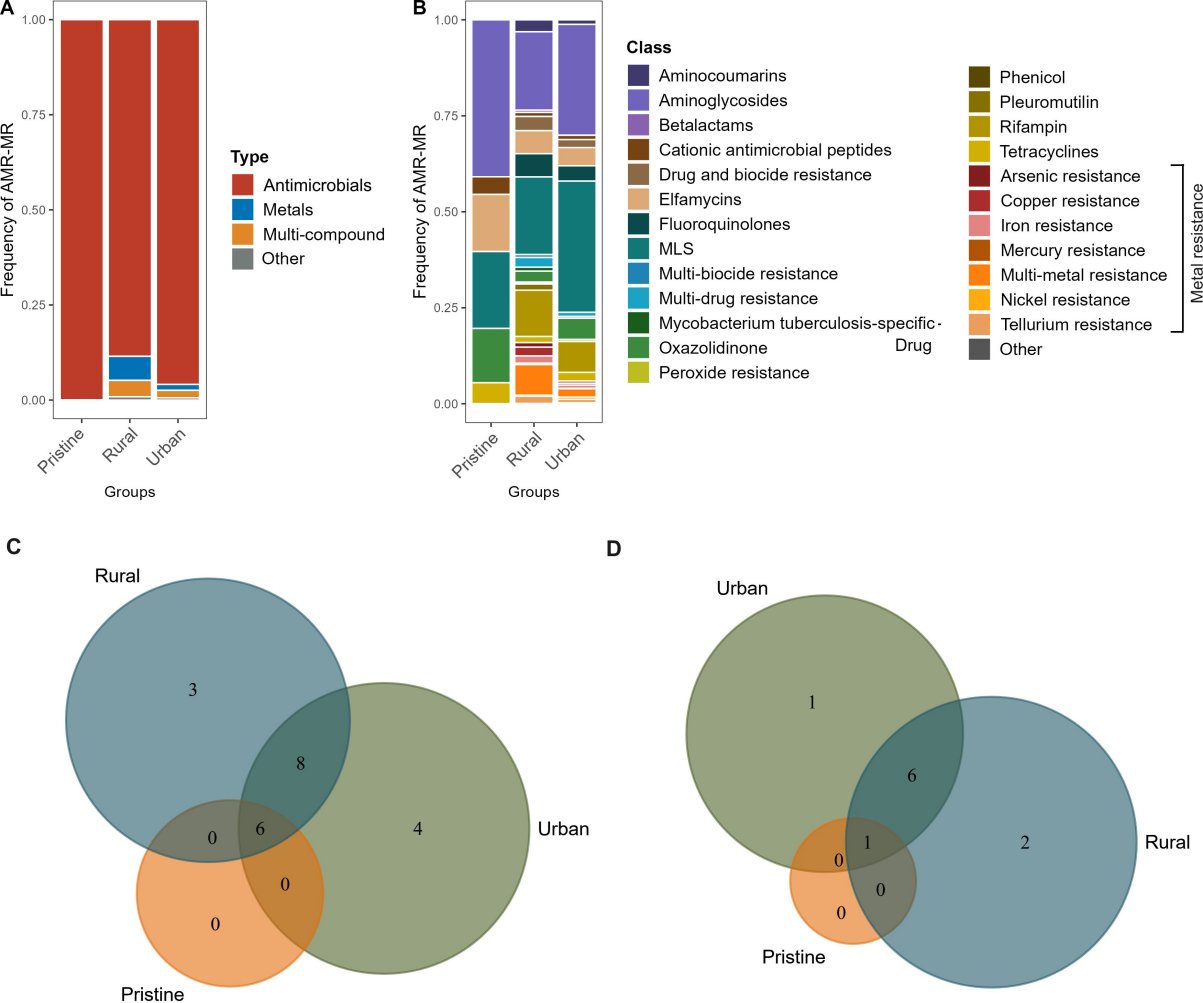
Overview of antimicrobial, metal, and biocide resistance in the pristine, rural, and urban environments. (**A**) Frequency of AMR–MR types in the three environments. (**B**) Frequency of AMR–MR classes in the three environments. Seven metal resistance classes are shown separately in the plot. (**C**) Distribution of AMR classes in the three environments. (**D**) Distribution of MR classes in the three environments.

The three major types of resistance identified across all environments were antibiotic resistance (AMR), metal resistance (MR), and multicompound resistance. In pristine samples, 99.9% of the resistance found was classified as AMR, with only 0.1% attributed to MR, and no multicompound resistance was detected. Aminoglycosides, elfamycins, oxazolidinone, and MLS accounted for more than 99% of the AMR detected in pristine samples. Rural samples showed a higher frequency of MR (10%) types compared to urban samples (4%) (Fig. 2A). Additionally, aminocoumarins, biocide resistance, fluoroquinolones, rifampin, and multidrug resistance were exclusively found in both rural and urban samples. Notably, metal and multimetal resistance were present in both rural and urban samples, with a frequency of 11% in rural samples compared to 2% in urban samples (Fig. 2B).

A total of 21 AMR classes were detected, with six being common across rural, urban, and pristine environments. These common classes included aminoglycosides, cationic antimicrobial peptides, elfamycins, MLS (macrolide–lincosamide–streptogramin), oxazolidinone, and tetracyclines. Among these, aminoglycosides and MLS were the most prevalent, making over 50% of the total frequency in all three environments. Elfamycins, oxazolidinone, and tetracycline were also present in all samples but at lower frequencies. Three AMR classes sulfonamides, glycopeptides, and fosfomycin, were exclusive to rural samples, while multidrug resistance, phenicol resistance, phenolic compound resistance, and pleuromutilin resistance were unique to urban samples (Fig. 2C).

In terms of MR, mercury resistance was common across all three environments, though it was found at a very low frequency in pristine samples (0.1%). In contrast, mercury resistance was more prevalent in rural (> 5%) and urban (> 2%) samples. No other MR class was detected in pristine samples. In rural and urban environments, resistance to iron, copper, nickel, arsenic, and tellurium was identified along with multimetal resistance. Metal and biocide resistance in combination and nickel resistance was exclusive to urban samples, while zinc resistance was specific to urban environments (Fig. 2D). These findings highlight the selective classes of AMR and MR and their relative abundance in environments with varying human impacts.

### AMR–MR indicators in pristine, rural, and urban environments

**Fig 3:**
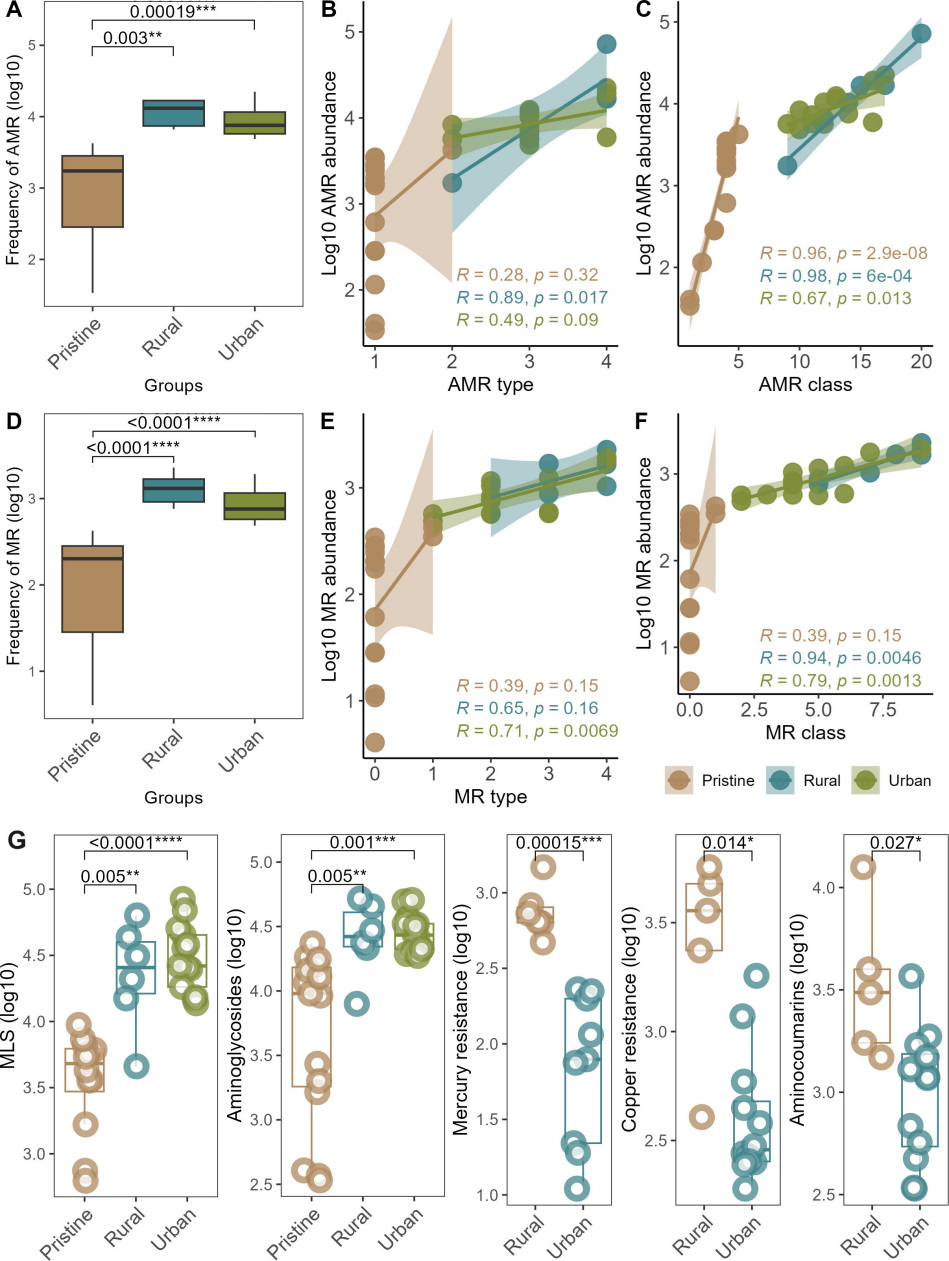
AMR–MR signatures in pristine, rural, and urban environments. (**A**) Variation in the AMR frequency across the three environments. (**B**) Regression analysis showing the correlation between AMR type and overall log-fold AMR abundance. (**C**) Regression analysis showing the correlation between AMR class and overall log-fold AMR abundance. (**D**) Variation in the MR frequency across the three environments. (**E**) Regression analysis showing the correlation between MR type and overall log-fold MR abundance. (**F**) Regression analysis showing the correlation between MR class and overall log-fold MR abundance. (**G**) Indicator AMR and MR classes in the three environments. *Significance at α-level of 0.05. **Significance at α-level of 0.005. ***Significance at α-level of 0.001.

The frequency of AMR in urban and rural samples was significantly higher (*P* < 0.005) compared to that of pristine samples (Fig. 3A). Only rural samples showed a significant correlation (*P* < 0.05) between AMR types and relative abundance (Fig. 3B). However, a significant correlation between AMR class and abundance was observed in pristine (*P* < 0.0001), rural (*P* < 0.001), and urban (*P* < 0.05) environments, indicating selective patterns in AMR classes across these environments. Pristine samples displayed a more significant correlation (*P* < 0.0001) than rural and urban samples due to the presence of fewer AMR classes and their corresponding lower abundance (Fig. 3C).

Similar to AMR, rural, and urban environments showed a significantly higher MR frequency compared to pristine samples (Fig. 3D). However, unlike AMR, urban samples demonstrated a positive correlation (*P* < 0.05) between the relative abundance and types of MR. In contrast, no significant correlation (*P* < 0.05) was observed between rural MR types and overall log-fold abundance (Fig. 3E). Notably, a positive correlation (*P* < 0.005) was found between MR class and abundance in both rural and urban samples (Fig. 3F).

Despite the higher frequency of MLS and aminoglycosides, their log-fold abundance was significantly lower in pristine samples compared to urban (*P* < 0.001) and rural (*P* < 0.005) environments. Rural samples also had a higher abundance of aminocoumarins (an AMR class) and two MR classes – mercury (*P* < 0.001) and copper (*P* < 0.05) resistance, compared to their urban counterparts (Fig. 3G). The difference in log-fold abundance for other AMR–MR classes was not significant (*P* > 0.05) (Fig. S5).

### Co-occurrence of AMR–MR and AMR–MR–MGE in pristine, rural, and urban environments

**Fig 4:**
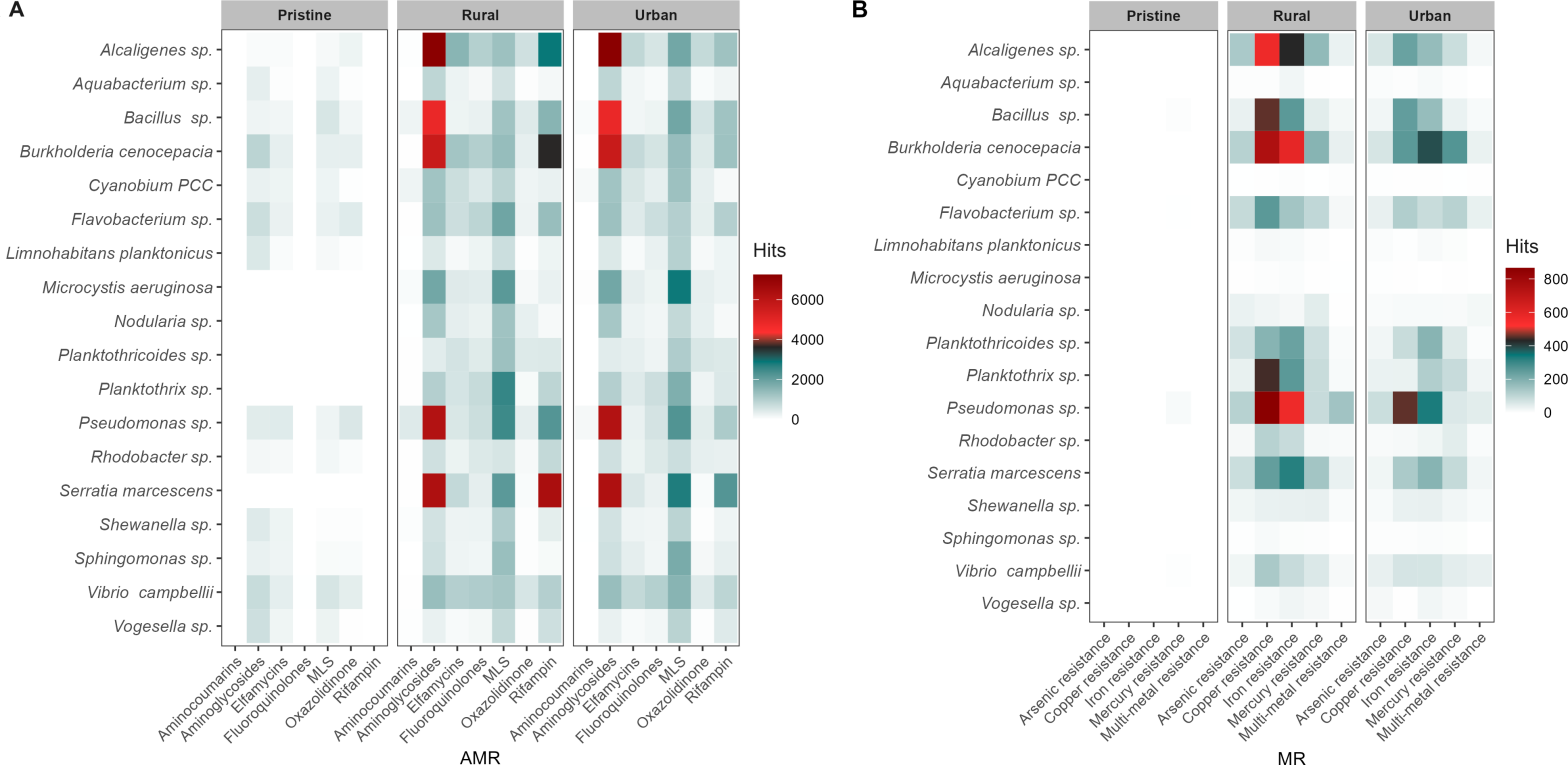
Species-specific AMR (**A**) and MR (**B**) profiles in pristine, rural, and urban environments. The heatmaps illustrate the most frequent AMR–MR classes (X-axis) across major species (Y-axis). The color gradient on the right indicates the number of AMR–MR hits. High- and medium-quality bins based on completion (>60%) and contamination (<20%) were selected for bacteria-specific AMR–MR classes.

In both rural and urban environments, *Burkholderia*, *Serratia*, *Bacillus,* and *Pseudomonas* exhibited the highest levels of AMR (Fig. 4A). Aminoglycosides and mercury resistance were found together in *Burkholderia* in more than 90% of rural and urban samples. Additionally, *Burkholderia* was predominantly associated with copper, iron, and mercury resistance, while *Pseudomonas* was also linked to iron and copper resistance in rural and urban samples. *Planktothrix* was primarily associated with copper resistance in rural samples. Among these, *Pseudomonas* was the only species with mercury resistance (0.1%) in the pristine environment (Fig. 4B).

Aminoglycoside, copper, and iron resistance co-occurred in *Burkholderia* and *Pseudomonas* in 95% of rural and 80% of urban samples (Fig. 4A and B). Aminoglycosides were the sole AMR class consistently found across all samples in rural and urban bacterial communities, highlighting their prevalence (Table S2).

**Fig 5:**
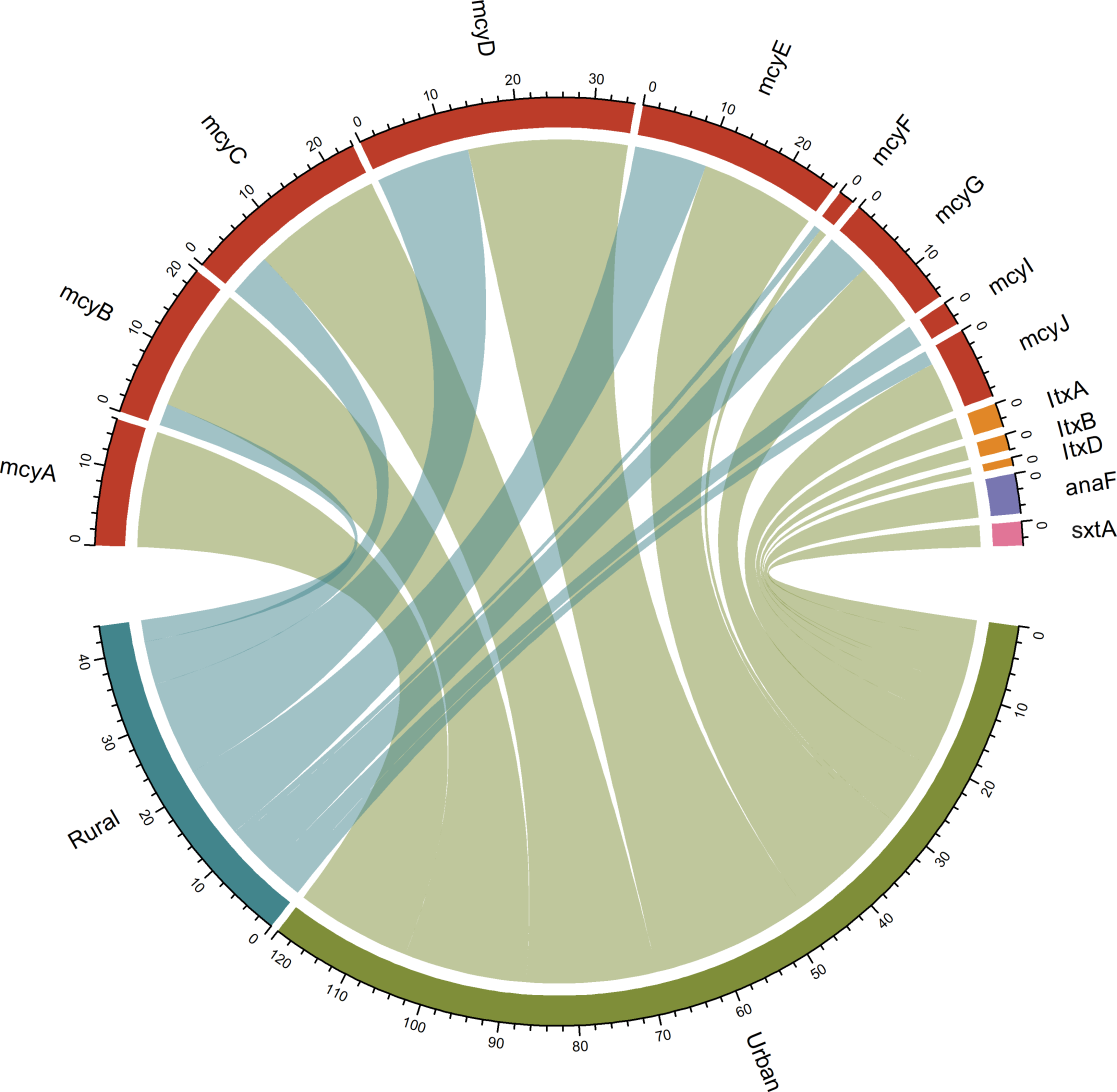
Chord diagram illustrating the copy number and distribution of cyanotoxin genes across three environments. The red arc represents microcystin genes from *Microcystis aeruginosa*, orange arc indicates lyngbya toxin (*ltx*) from *Lyngbya majuscule,* and purple and pink arcs signify anatoxin (*ana*F) from *Anabaena* sp. saxitoxin from *Cylindrospermopsis raciborskii*.

Interestingly, despite the low frequencies of AMR and MR in Cyanobacteria, they were rich in toxin genes including microcystin gene clusters (*mcy*A–E), lyngbya toxins (*Ltx*A, *Ltx*B, and *Ltx*D) anatoxin (*Ana*F), and saxitoxin (*Sxt*A). In contrast, pristine samples remained free of MR and toxins, underscoring the purity of aquatic environments with minimal human impacts (Fig. 4B and 5).

**Fig 6:**
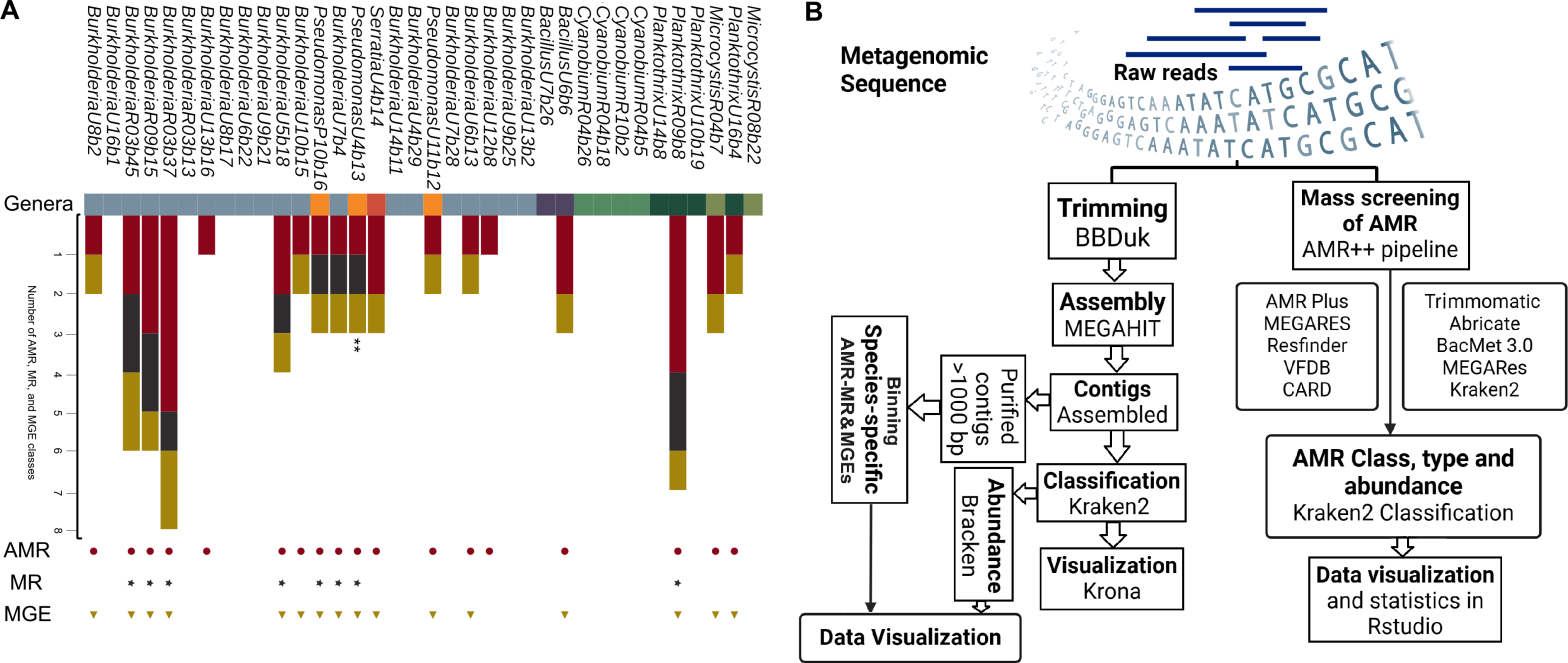
An overview of the co-occurrence of the methodology. (**A**) Phylogenetic tree representing the co-occurrence of antimicrobial resistance (AMR), metal resistance (MR), and mobile genetic elements (MGE) in pristine, rural, and urban environments. The GTOtree program was used to create this tree and based on identifying coding sequences (prodigal) and target genes (hmmer3) from high- and medium-quality genomes (CheckM) and classification with kraken2. The vertical bar with different colors indicates specific genera in the tree. The multicolor horizontal bar represents the number of positive hits for AMR and MR classes and MGEs. Red circular dots, gray stars, and gold triangles indicate the presence of AMR, MR, and MGEs in the classified bins, respectively. **Positive for the *int*I1 gene. (**B**) Diagram illustrating the research design and techniques for detecting antimicrobial resistance (AMR), metal resistance (MR), and mobile genetic elements (MGEs) in pristine, rural, and urban environments.

We further selected 36 high- and medium-quality bins in terms of completeness (> 80%), contamination (<20%), and heterogeneity (> 0.5%) (Table S3) for further screening of AMR, MR, and mobile genetic elements (MGEs). *Burkholderia*, *Pseudomonas,* and *Planktothrix* were associated with AMR–MR–MGE co-occurrence in rural, urban, and pristine environments. High-quality bins for *Burkholderia* (*n* = 3) and *Planktothrix* (*n* = 1) were positive for aminoglycoside, mercury resistance, and transposons. Significantly, AMR–MR–MGE co-occurrence was also identified in one medium-quality *Pseudomonas* bin from the pristine environment. AMR–MR co-occurrence was also identified in *Bacillus* and *Microcystis* bins in the urban and rural environments. One *Pseudomonas* bin from the urban site was also positive for anthropogenic marker gene integron–integrase (*int*I1) from *P. aeruginosa* (Fig. 6A).

## DISCUSSION

Human activities play a significant role in the spread of antimicrobial resistance (AMR) and metal resistance (MR). Yet, how anthropogenic activities impact the prevalence of AMR–MR remains largely unknown. We identified microbial communities and quantified AMR–MR abundance in pristine, rural, and urban aquatic environments. Pristine samples are characterized by low AMR–MR abundance, low levels of pathogens, and the absence of toxigenic cyanobacteria, indicating minimal or no impacts from human activities.

Certain genera, such as *Limnohabitans*, *Sphingomonas,* and *Rhodobacter,* were consistently detected in all samples, representing normal flora in aquatic environments (30, 31). Pristine samples predominantly consisted of Actinobacteria and Alphaproteobacteria, including *Acetobacter*, *Streptomyces*, *Aquabacterium*, *Limnohabitans*, *Rhodobacter*, *Vogesella*, *Caulobacter,* and *Cerelibacter*. These bacteria are considered nontoxic and harmless in aquatic environments (32, 33). In rural and urban environments, most of the bacteria present are contaminating microbes such as Cyanobacteria, *Shewanella*, and *Vibrio* (34, 35) and biotic stress adapters and metal-thriving species from *Bacillus* and *Pseudomonas* (36). Despite their low abundance in the communities, these latter groups were associated with high frequencies of AMR–MR in rural and urban environments. This suggests potential metal contamination resulting from a complex interplay of extrinsic and intrinsic factors, such as agricultural practices, recreational activities, floods, water runoff, and aquatic pollution. More concerningly, some AMR and MR from *Bacillus* and *Pseudomonas* were also linked to mobile genetic elements (MGEs), indicating the potential for resistance transfer in aquatic environments. Conversely, the homogeneity of AMR classes and the contamination-free environment with nearly zero MR further emphasize the purity of the pristine environment.

Highly dominant Cyanobacteria (*Cyanobium*, *Microcystis*, and *Planktothrix*) differentiated the pristine samples from the rural and urban counterparts regarding microbial communities. While the pristine environment is relatively free of cyanobacteria (0.4%), it was the most and second richest dominant bacteria in the rural and urban samples. Cyanobacteria have been reported as potential reservoirs for AMR in aquatic habitats (26, 37). Specifically, during the bloom season in summer and autumn, cyanobacteria, mainly *Microcystis aeruginosa*, *Planktothrix agardhii*, *Nostoc* sp., and *Anabaena* sp., interact with other bacteria that harbor AMR in aquatic habitats (38). Our data show that four major toxigenic cyanobacteria*—Microcystis aeruginosa*, *Planktothrix* sp., *Plankthricoides* sp., and *Nodularia* sp.—were correlated with certain classes of AMR in these two environments, while the species were absent in the pristine environment. Additionally, highly abundant *Microcystis* and *Planktothrix* were associated with potentially health-concerning cyanotoxins, alongside iron and copper resistance in rural and urban samples.

This study identified several AMR–MR classes with CARD, Resfinder, ARG-ANNOT, and AMRFinderPlus using the AMR ++ pipeline and the MEGARES database (v3). The pipeline is highly specialized for the characterization of AMR–MR from metagenomic data sets (39). Ten AMR–MR classes were identified in both rural and urban samples. Although comparative metagenomic studies for AMR in aquatic environments in Australia are not yet available, the observed number appears relatively high compared to results reported from similar environments in Bangladesh, China, and India (40–42). However, metal and biocide resistance in rural and urban environments is consistent with previous studies in Australia (43, 44).

Our findings indicate that metal, multimetal, and multicompound resistance are prevalent in rural samples, suggesting contamination from external sources or effluents. The semi-rural region of Raymond Terrace, which includes agricultural lands and animal farms, appears to be a significant source of MR, consistent with previous studies (45, 46). Notably, heavy rains and flooding were reported in Raymond Terrace and greater Sydney before sample collection in 2020 compared to previous 5 years, leading us to hypothesize that the increased prevalence of metal and multicompound resistance may result from agricultural waste contamination from rainwater runoff (47).

In contrast, pristine samples were relatively free of MR, except for the mercury resistance class. Interestingly, none of the contigs and bins from the pristine samples exhibited a BLAST similarity hit score exceeding 95% for MR and cyanotoxin genes against our customized database. The mercury resistance gene, *mer*A, was detected in pristine samples only by lowering the similarity threshold score to 90%. For rural and urban samples, the threshold was set at 99%, as setting it at 95% resulted in 1,586 hits. This lower genomic relatedness of *mer*A in pristine samples suggests a potentially ancient trait and underscores the impact of anthropogenic activities on the selection of MR in aquatic environments.

We next investigated the relative abundance of AMR–MR and observed trends across pristine, rural, and urban environments. In line with previous research that linked a proportion of the total resistome with specific AMR–MR phenotypes (48–50), pristine samples exhibited lower diversity in AMR classes compared to rural and urban samples, which showed greater heterogeneity. Distinctly, the microbial community in pristine environments lacks Cyanobacteria and opportunistic pathogens such as *Serratia*, resulting in low and homogenous AMR classes and nearly zero MR. However, *Bacillus*, *Burkholderia*, and *Pseudomonas* were linked to high frequencies of AMR–MR in both rural and urban environments. Among specific bacterial species, *A. faecalis*, *Burkholderia* sp.*, B. pumilus*, *P. putida*, *P. alcaligenes*, and *P. aeruginosa* have been reported to harbor much of the MR in the environment (51, 52). *Bacillus* and *Pseudomonas* strains actively take up heavy metals and potentially contribute to the remediation of mutimetals and multicompounds (53, 54). Specifically, previous studies have demonstrated that *Pseudomonas* species dominate microbial communities, with many exhibiting co-selection potentials for multimetal and multidrug resistance in metal-contaminated sites (55) and freshwater reserves (rivers and lakes) (56). These studies reported that agricultural, aquacultural, and recreational activities accelerate the co-occurrence of AMR–MR in soil and aquatic environments. *P. fluorescens* and *P. aeruginosa* were also reported to harbor mobile genetic elements (MGEs) even in pristine samples (57, 58). Consistent with previous studies, the presence of *Pseudomonas* in both rural and urban water, along with the richness of MR, suggests contamination by heavy metals due to human activities such as agricultural practices in rural and recreational activities in urban settings.

In this study, we observed *Burkholderia* and *Pseudomonas*-associated aminoglycoside and mercury resistance co-occurrence in most samples. This co-occurrence was prevalent in all three environments, including *Pseudomonas* in pristine samples. The frequent co-occurrence of aminoglycoside and mercury resistance is associated with an integron–integrase gene called *int*I1, a potential genetic marker of anthropogenic pollution (59). Previous environmental studies have revealed a strong association between AMR and the *int*I1 gene (60). A higher prevalence of *int*I1 promotes the rapid dissemination of both AMR and MR, reflecting the extent of anthropogenic activities in aquatic environments (61). Additionally, iron and copper resistances play a co-selective role in aminoglycoside, beta-lactam, sulfonamide, or tetracycline resistance (62). Notably, the majority (80%) of *Burkholderia* and *Pseudomonas* species exhibited co-occurrence of aminoglycoside and multimetal resistance, emphasizing the impact of iron and copper in shaping resistance patterns in rural and urban aquatic environments. The genus *Burkholderia* was previously classified under *Pseudomonas*, and both have similar functions in aquatic environments (63). In this study, at least 80% of the *Burkholderia* bins were separated from *Pseudomonas* by a slight margin in similarity percentages. We can postulate that microbes with similar genome organization and functions are mostly associated with AMR–MR dissemination via MGEs. A bacterial strain with co-occurring multidrug and multimetal resistance poses greater threats to the environment and human health than drug–drug, metal–metal, or drug–metal pairs (64). The type of co-occurrence observed in bacteria within rural and urban environments raises concerns for public health safety and environmental sustainability. This concern makes proper environmental and water management plans essential for all water bodies.

We report the prevalence of AMR–MR in pristine, rural, and urban water bodies. Further research should identify specific factors such as temperature, population density, agricultural practice, environmental contaminants, sanitation, drugs, and their disposals contributing to the dissemination of AMR–MR and their co-occurrence in bacterial genera and transposons. The potential health risks to humans, animals, and environments from the AMR–MR–MGE relationship, drug–metal interactions, and toxin-producing bacteria in Australian rural and urban areas highlight the need for sustainable water management in lakes, reserves, and rivers. Given the correlation between drug and multi-compound resistance in rural environments, farming and aquaculture practices must be conducted with greater care and control to prevent contamination. Our study’s rural samples were collected 6 years after the urban and pristine samples. The comparison of AMR/MR is valid, considering the static population, ecology, and level of human activity in the rural sample area during the sampling period. All samples underwent processing and sequencing from the same center, along with consistent data generation and analysis. However, we recommend year-round long-term survey studies to monitor the AMR–MR dynamics over time, including interactions via horizontal gene transfer and transposable elements.

## MATERIALS AND METHODS

### Sample collection, processing, and data curation

Metagenomic samples were collected from three distinct Australian sites during the summer and autumn. For the urban site, 17 samples were collected from Kensington Pond, Centennial Parkland, Sydney, NSW (−33.8922 S 151.2322 E) from December–April (2014–2015), in support of the NSW Office of Water. Six samples were collected between December and May (2020–2021) from Ross Wallbridge Reserve, Raymond Terrace (32.7573 S, 151.7482 E), a rural site in NSW, Australia. Samples were collected in sterile 500 mL bottles and transported (<2 hours) to the laboratory by maintaining a cold chain (4°C). Samples were processed by filtration of 100 mL water in 0.22-µm glass filters. Following the manufacturer’s instructions, DNA extraction was performed with the FastDNA Spin Kit for soil (MP Biomedicals, USA), followed by quality checking in a spectrophotometer (Thermo Fisher Scientific, USA). Metagenomic library preparation and sequencing were performed by the Ramaciotti Centre for Genomics (The University of New South Wales, UNSW, Australia). For the pristine data set, 16 metagenome data sets from Shark Bay, Western Australia (25.7834 S, 113.2988 E) were curated from the National Centre for Biotechnology Information (NCBI) in April 2014 (65). Previous studies have shown this site to be largely anthropogenic pollution-free (66, 67).

### Shotgun metagenome assembly and annotation

The generated paired-end reads were concatenated before processing. To maintain uniformity and attain minimum depth for AMR screening, four samples (three from Shark Bay and one from Centennial Park) with less than 75 million reads were discarded. FastQC was used to check the quality of reads before and after trimming (68). Illumina adapters, short synthetic bases, and low-quality bases were trimmed and filtered with bbduk (v39.06) (69). The metagenomic assembly was performed using MEGAHIT (v1.2.9) with default parameters (70). The error correction tools were employed, and contigs less than 1,000 bp were discarded. Quality assessment of the assembly was performed using Quast (v5.2.0) (71) and assembly-stat program (https://github.com/sanger-pathogens/assembly-stats). Read classification was performed using Kraken2 (v2.0.8) (72), and Bayesian Re-estimation of Abundance with Kraken (Bracken) was used to compute the abundance of different taxa (73). Reads were mapped to each sample using Bowtie2 (v2.5.1) to generate coverage information (74). Sorting and indexing of mapped reads were performed using SAMtools (v1.16.1) (75). The details of reads, assembly, and classification are in Table S1. Taxonomic data were visualized as circular plots using Krona (76) and revised further in phyloseq (77) and NetworkD3 (78) R packages. Singletons were removed, and only abundant taxa (phylum, genera, and species) were considered for further analysis.

### Identification of AMR and MR

Metagenomic reads were screened using AMR ++ in conjunction with MEGARes (v3) to detect antimicrobial resistance (AMR) and heavy metal resistance (MR) (39). The standard AMR Nextflow pipeline, which is part of the conda profile, was utilized for AMR and MR detection. The pipeline includes trimming, host removal, resistome profiling, AMR–MR hit detection, and classification (types, classes, and mechanisms of action). This pipeline has been developed to support a variety of databases including NCBI, Comprehensive Antibiotic Resistant Database (CARD), Antibiotic Resistant Gene ANNOTation (ARG-ANNOT), Resfinder, MEGARES, ECoH, PlasmidFinder, and The Virulence Factor Database (VFDB). The bioinformatics workflow of the study is schematically represented in Fig. 6B.

### Identification of AMR-, MR-, and MGE-associated bacteria

Following the MEGAHIT assembly, contigs shorter than 1,000 bp were discarded to prevent ambiguous annotation. Metagenomic binning was performed with MetaBAT2 (v2.15) and MaxBin2 (v2.2.7) (79, 80). The resulting bins were further purified using MAGpurify (v2.1.2) (81) and dereplicated using DAS tools (v1.1.6) (82). Bin quality, coverage, and contamination were verified using CheckM (v1.2.2) (83). Only high-quality (>90 completeness, <10% contamination) and medium-quality (60–90 completeness, 10%–20% contamination) 36 bins were selected as metagenome-assembled genomes (MAGs) for further AMR–MR–MGE analysis. These selected bins were taxonomically classified using the Kraken2 microbial database (72). Based on completion and contamination, high- and medium-quality purified classified *Bacillus*, *Burkholderia*, *Cyanobium*, *Microcystis*, *Pseudomonas,* and *Planktothrix* bins were screened for multiresistance and AMR–MR co-occurrence using CARD, ARG-ANNOT, Resfinder, and MEGARES. Additionally, these bins were screened further for mobile genetic elements (MGEs) associated with horizontal gene transfer (HGT) against the customized HGT database as described previously (84). We used BLASTn to compare our genome against seven comprehensively compiled ARG databases for transferable ARGs.

### Statistical analysis

A one-way ANOVA with Tukey’s *post-hoc* test was used to compare the mean AMR–MR frequency and classes across pristine, rural, and urban samples. The correlation between the type/class of AMR–MR and its abundance was examined using the regression correlation coefficient with corr R packages. A *p*-value of <0.05 was considered statistically significant throughout all data analysis stages.

## Data Availability

Raw metagenomic sequence data are publicly available at the National Center for Biotechnology Information (NCBI) under the BioProject accession numbers PRJNA429237 (pristine), PRJNA1179635 (rural), and PRJNA1095672 (urban). Metagenomic assemblies, bins (MAGs), and scripts for data and statistical analysis are accessible from the GitHub repository via https://github.com/FoysalRon/PRU.

## SUPPLEMENTAL MATERIAL

Supplemental material aem.02317-24-s0001.docx Tables S1 to S3; Figures S1 to S5.

ASM does not own the copyrights to Supplemental Material that may be linked to, or accessed through, an article. The authors have granted ASM a non-exclusive, world-wide license to publish the Supplemental Material files. Please contact the corresponding author directly for reuse.
