# Supplementary material for: The impact of anthropogenic activities on antimicrobial and heavy metal resistance in aquatic environments
Source: Appl Environ Microbiol. 2025 Mar 12;91(4):e02317-24. doi: 10.1128/aem.02317-24 (PMC12016542; doi:10.1128/aem.02317-24)
Supplement: Supplemental material — Tables S1 to S3; Figures S1 to S5. [file aem.02317-24-s0001.docx]

**The impact of anthropogenic activities on antimicrobial and heavy metal resistance in aquatic environments**

**Running title: Antimicrobial Resistance in Aquatic Environments**

Md Javed Foysal, Brett Neilan, Verlaine Timms

School of Environmental and Life Sciences, The University of Newcastle, Callaghan, NSW 2308, Australia

Australian Research Council Centre of Excellence in Synthetic Biology, Australian Research Council, Macquarie Park, NSW 2109, Australia

ORCID

Md Javed Foysal: 0000-0002-2064-8897

Verlaine Timms: 0000-0002-3883-4428

Brett Neilan: 0000-0001-6113-772X

*Corresponding author:

Brett A. Neilan

Email: brett.neilan@newcastle.edu.au

Funding Information

This research was conducted by the Australian Research Council Centre of Excellence in Synthetic Biology (CE200100029) and funded by the Australian Government.

Table S1. Samples and sequence statistics for 35 metagenomic datasets

| Samples | Groups | Reads | N50 | N75 | N90 |
| --- | --- | --- | --- | --- | --- |
| A4 | Urban | 88,236,244 | 533 | 422 | 389 |
| A5 | Urban | 87,956,832 | 463 | 376 | 332 |
| A6 | Urban | 87,6432,78 | 540 | 424 | 391 |
| A7 | Urban | 78,688,362 | 449 | 372 | 330 |
| A8 | Urban | 77,238,439 | 448 | 360 | 324 |
| A9 | Urban | 72,456,786 | 423 | 359 | 324 |
| A10 | Urban | 87,984,336 | 515 | 421 | 390 |
| A11 | Urban | 85,643,287 | 562 | 426 | 391 |
| A12 | Urban | 85,292,236 | 524 | 420 | 389 |
| A13 | Urban | 84,887,363 | 638 | 427 | 389 |
| A14 | Urban | 76,232,564 | 526 | 412 | 385 |
| A15 | Urban | 77,186,426 | 562 | 419 | 388 |
| A16 | Urban | 74,873,671 | 531 | 412 | 386 |
| A17 | Urban | 86,425,321 | 572 | 419 | 387 |
| A18 | Urban | 83,843,876 | 566 | 417 | 387 |
| A19 | Urban | 74,880,031 | 469 | 405 | 382 |
| A20 | Urban | 81,565,634 | 513 | 422 | 390 |
| A21 | Urban | 76,633,843 | 457 | 411 | 386 |
| A22 | Urban | 86,674,238 | 539 | 425 | 390 |
| A23 | Urban | 92,122,533 | 523 | 423 | 390 |
| A24 | Urban | 78,787,023 | 513 | 412 | 387 |
| RW04 | Rural | 82,668,116 | 527 | 402 | 324 |
| RW05 | Rural | 78,871,781 | 498 | 398 | 312 |
| RW06 | Rural | 82,892,291 | 509 | 407 | 342 |
| RW07 | Rural | 79,243,096 | 488 | 438 | 351 |
| RW08 | Rural | 82,249,241 | 526 | 467 | 372 |
| RW09 | Rural | 84,821,271 | 536 | 478 | 376 |
| SB01 | Pristine | 83,596,064 | 671 | 440 | 348 |
| SB02 | Pristine | 80,023,916 | 936 | 517 | 378 |
| SB03 | Pristine | 86,886,671 | 1030 | 532 | 381 |
| SB04 | Pristine | 74,582,646 | 782 | 452 | 381 |
| SB05 | Pristine | 82,483,215 | 886 | 512 | 392 |
| SB06 | Pristine | 72,981,032 | 561 | 436 | 372 |
| SB07 | Pristine | 86,223,091 | 692 | 489 | 391 |
| SB08 | Pristine | 82,456,981 | 489 | 476 | 365 |
| SB09 | Pristine | 83,562,221 | 587 | 467 | 368 |
| SB10 | Pristine | 98,886,336 | 1002 | 502 | 394 |
| SB11 | Pristine | 78,231,765 | 538 | 418 | 312 |
| SB12 | Pristine | 82,776,782 | 588 | 496 | 372 |
| SB13 | Pristine | 84,343,331 | 602 | 511 | 386 |
| SB14 | Pristine | 81,562,781 | 556 | 478 | 376 |
| SB15 | Pristine | 79,991,223 | 501 | 436 | 371 |
| SB16 | Pristine | 82,562,891 | 536 | 472 | 338 |

Table S2. Co-occurrence of AMR-MR classes, presented as % bins, in pristine, rural, and urban environments

| **AMR class** | Pristine | Rural | Urban |
| --- | --- | --- | --- |
| Aminoglycosides | 67 | 100 | 100 |
| Aminocoumarins | 9 | 32 | 28 |
| Elfamycins | 18 | 56 | 45 |
| MLS | 48 | 95 | 95 |
| Oxazolidinone | 18 | 67 | 78 |
| Fluoroquinolones | 2 | 32 | 22 |
| Rifampin | 2 | 78 | 56 |
| Multi-drug resistance | 7 | 12 | 9 |
| Drug and biocide resistance | 0 | 6 | 2 |
| **MR-class** |  |  |  |
| Iron resistance | 0 | 26 | 21 |
| Copper resistance | 0 | 24 | 14 |
| Mercury resistance | 1 | 78 | 52 |
| Multi-metal resistance | 0 | 20 | 15 |

Table S3. High and medium-quality bins selected in this study for co-occurrence analysis.

| **Bins** | Source | Marker | Completeness | Contamination | Heterogeneity |
| --- | --- | --- | --- | --- | --- |
| P10b16 | Pristine | *Pseudomonas* | 82.4 | 4.6 | 3.5 |
| R03b13 | Rural | *Burkholderia* | 94.8 | 0.6 | 2.8 |
| R03b37 | Rural | *Burkholderia* | 82.5 | 3.2 | 4.6 |
| R04b5 | Rural | *Cyanobium* | 93.5 | 7.8 | 5.2 |
| R04b7 | Rural | *Microcystis* | 80.2 | 6.7 | 2.8 |
| R04b18 | Rural | *Cyanobium* | 72.1 | 5.8 | 2.2 |
| R04b26 | Rural | *Cyanobium* | 89.3 | 3.1 | 5.6 |
| R08b22 | Rural | *Microcystis* | 93.5 | 0.8 | 4.9 |
| R09b8 | Rural | *Planktothrix* | 92.1 | 2.6 | 2.1 |
| R09b15 | Rural | *Burkholderia* | 73.4 | 8.4 | 0.9 |
| U4b13 | Urban | *Pseudomonas* | 70.3 | 3.5 | 12.5 |
| U4b14 | Urban | *Serratia* | 60.1 | 19.9 | 15.5 |
| U4b29 | Urban | *Burkholderia* | 68.0 | 4.9 | 1.2 |
| U5b18 | Urban | *Burkholderia* | 71.4 | 1.4 | 7.4 |
| U6b6 | Urban | *Bacillus* | 63.8 | 7.2 | 1.5 |
| U6b13 | Urban | *Burkholderia* | 63.1 | 4.6 | 1.2 |
| U6b22 | Urban | *Burkholderia* | 68.6 | 2.2 | 2.8 |
| U7b4 | Urban | *Burkholderia* | 69.1 | 10.2 | 9.6 |
| U7b26 | Urban | *Bacillus* | 63.5 | 7.6 | 0.5 |
| U7b28 | Urban | *Burkholderia* | 66.8 | 3.7 | 0.9 |
| U8b2 | Urban | *Burkholderia* | 82.2 | 15.5 | 3.9 |
| U8b17 | Urban | *Burkholderia* | 65.5 | 5.3 | 1.9 |
| U9b21 | Urban | *Burkholderia* | 64.8 | 1.6 | 0.7 |
| U9b25 | Urban | *Burkholderia* | 56.9 | 4.6 | 0.5 |
| U10b15 | Urban | *Burkholderia* | 71.7 | 9.3 | 1.1 |
| U10b19 | Urban | *Planktothrix* | 76.7 | 6.0 | 0.8 |
| U11b12 | Urban | *Burkholderia* | 63.9 | 9.8 | 3.3 |
| U12b8 | Urban | *Burkholderia* | 67.3 | 6.0 | 1.2 |
| U13b2 | Urban | *Burkholderia* | 69.4 | 7.6 | 1.1 |
| U13b16 | Urban | *Burkholderia* | 69.0 | 5.9 | 6.8 |
| U14b8 | Urban | *Planktothrix* | 92.7 | 19.7 | 7.2 |
| U14b11 | Urban | *Burkholderia* | 65.6 | 12.7 | 12.5 |
| U16b1 | Urban | *Burkholderia* | 67.9 | 6.7 | 2.5 |
| U16b4 | Urban | *Planktothrix* | 71.8 | 16.4 | 30.0 |


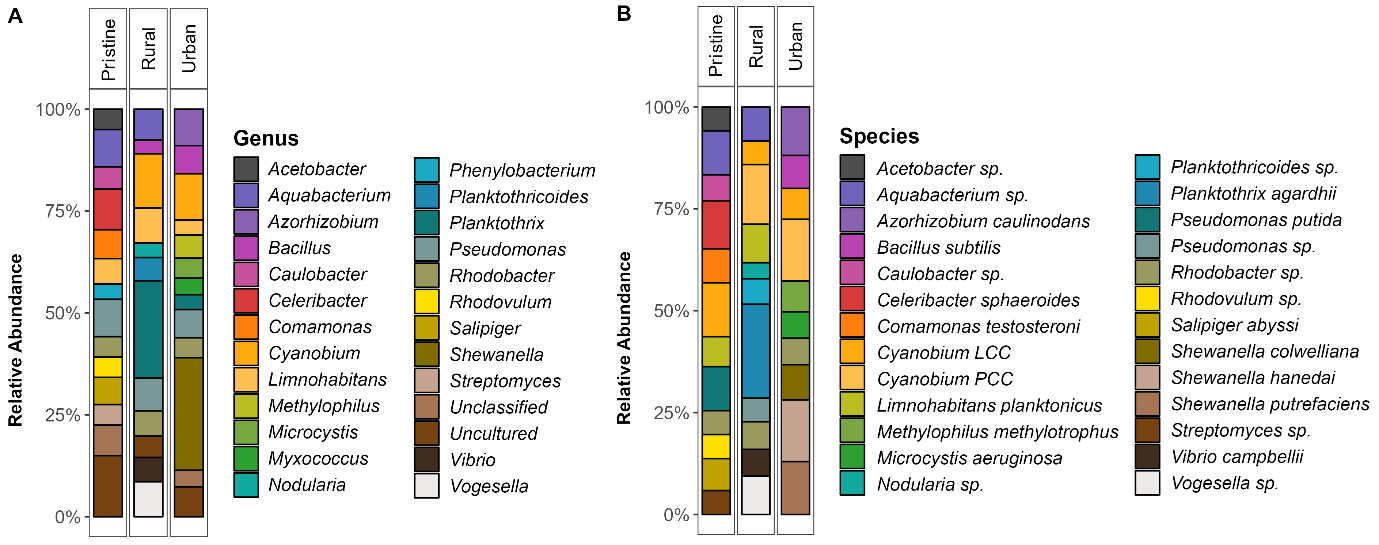


**FIG S1** Top 26 genera (A) and species (B) in pristine, rural, and urban environments. The bar charts show genera and species with more than 3% and 2% relative abundance in each environment.


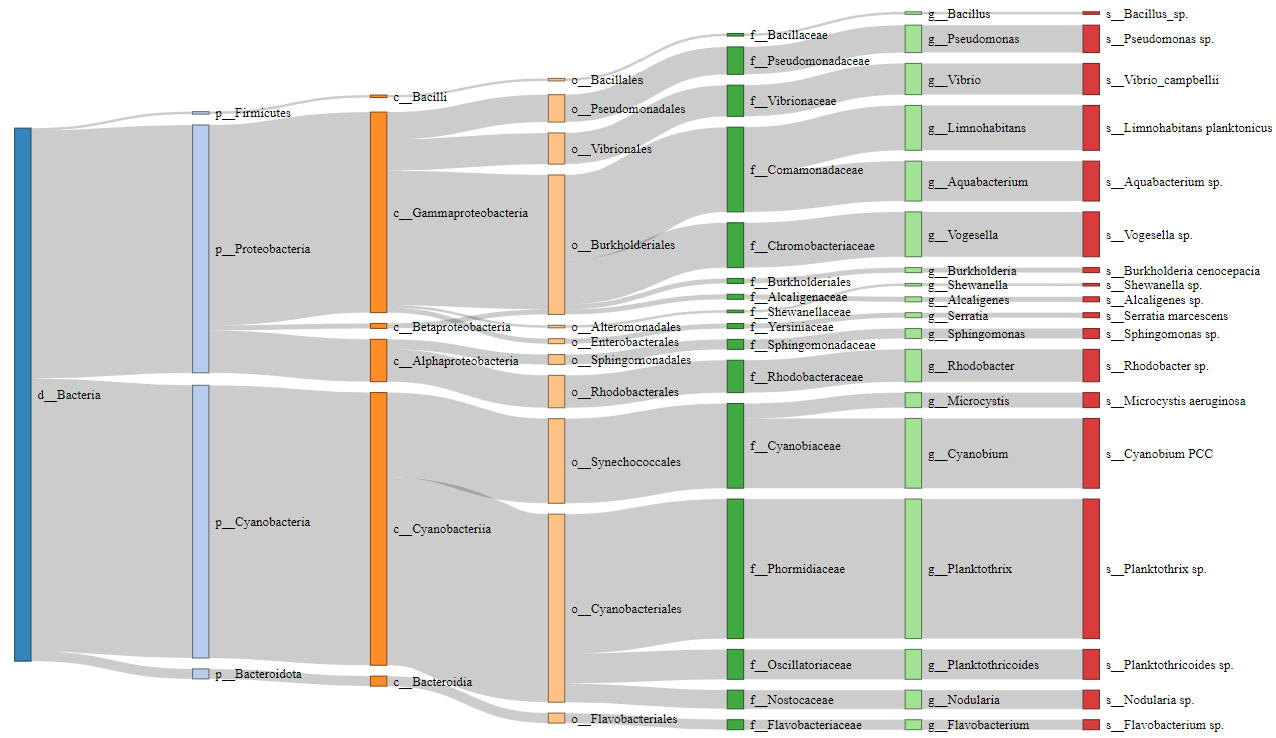


**FIG S2** Snaky plot depicting microbial community abundance at various taxa levels in rural samples.


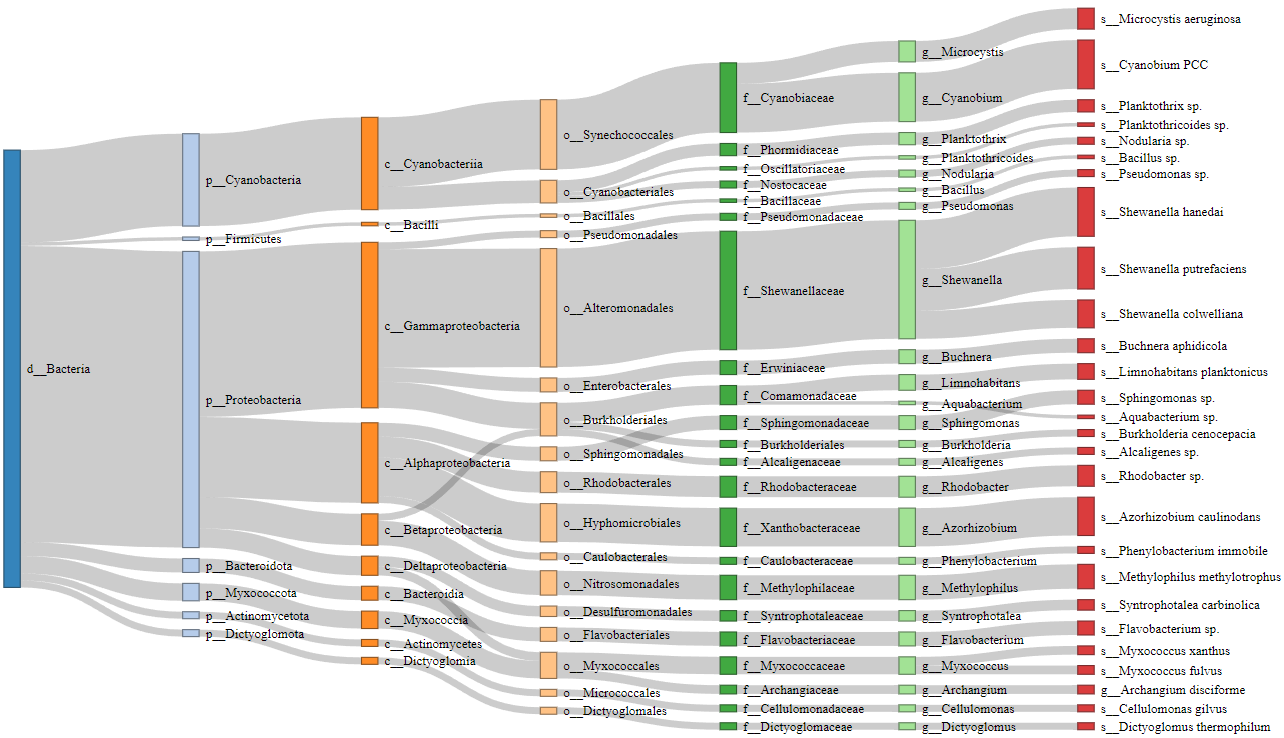


**FIG S3** Snaky plot depicting microbial community abundance at various taxa levels in urban samples.


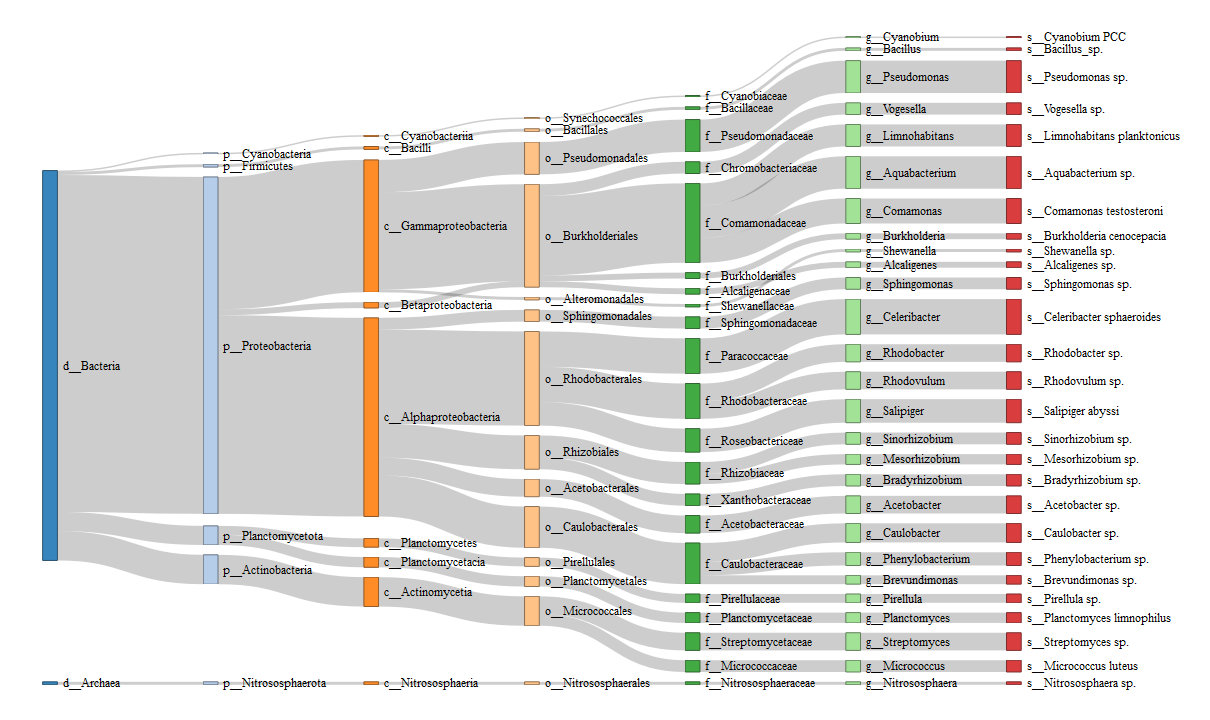


**FIG S4** Snaky plot depicting microbial community abundance at various taxa levels in pristine samples.


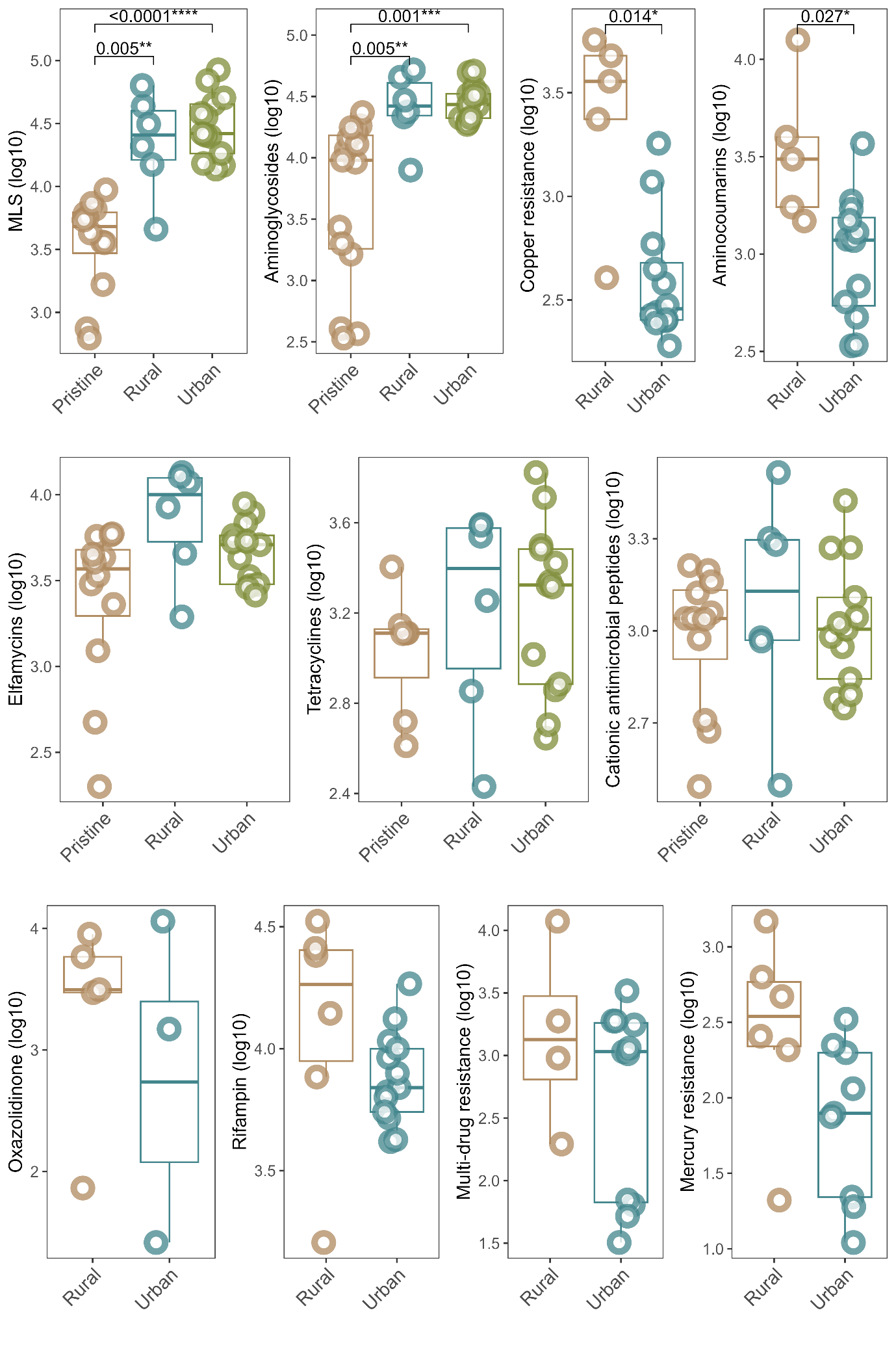


**FIG S5** An overview of the differential abundance of AMR across three aquatic environments.
